# Supplementary material for: Structural Insights into Endostatin–Heparan Sulfate Interactions Using Modeling Approaches
Source: Molecules. 2024 Aug 26;29(17):4040. doi: 10.3390/molecules29174040 (PMC11397277; doi:10.3390/molecules29174040)
Supplement: Supplementary file 1 [file molecules-29-04040-s001.zip › Supplementaries_Uciechowska_R1.pdf]

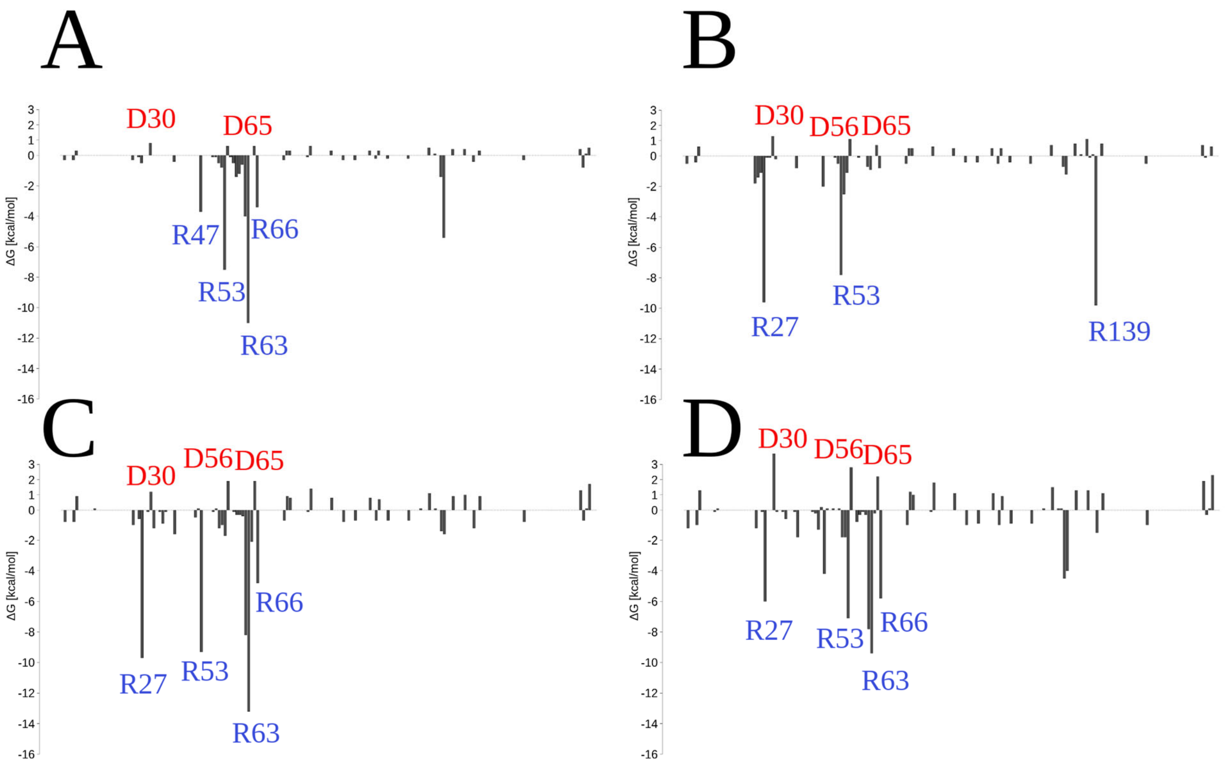

Figure S1. Per residue free energy decomposition for the last nanosecond of the productive MD run for HP $^{1}C_4$ dp2 (A), dp4 (B), dp6 (C) and dp8 (D). In blue and red are amino acid residues showing the most favourable and unfavourable effect on binding energy, respectively.

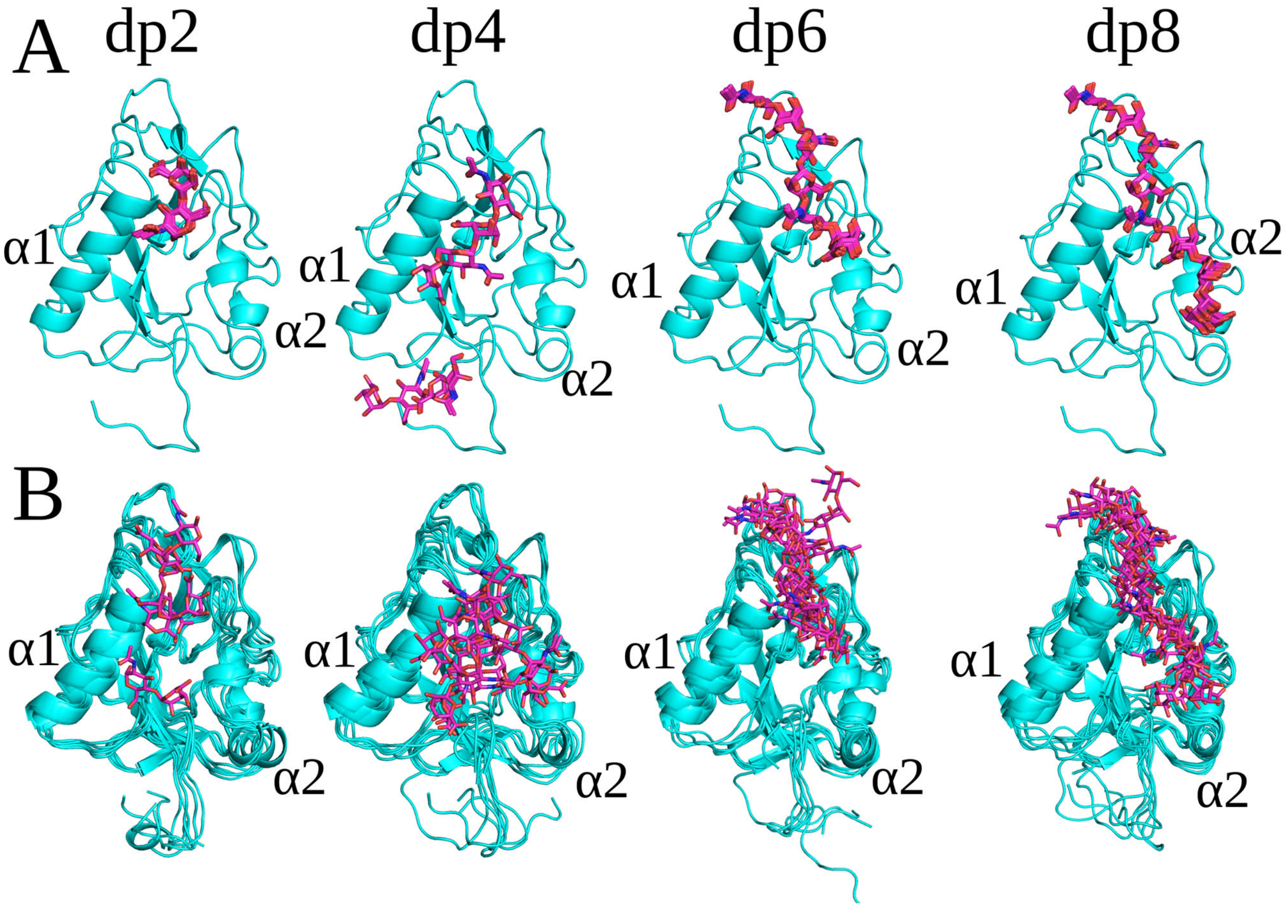

Figure S2. deHP dp2, dp4, dp6 and dp8 top 50 docking poses (endostatin – blue colour, cartoon; HP – pink colour, licorice) (A); Structures of the complexes obtained from the last frame in MD

simulations (endostatin – blue colour, cartoon; HP: cluster 1 – pink colour, cluster 2 – yellow colour; licorice) (B).

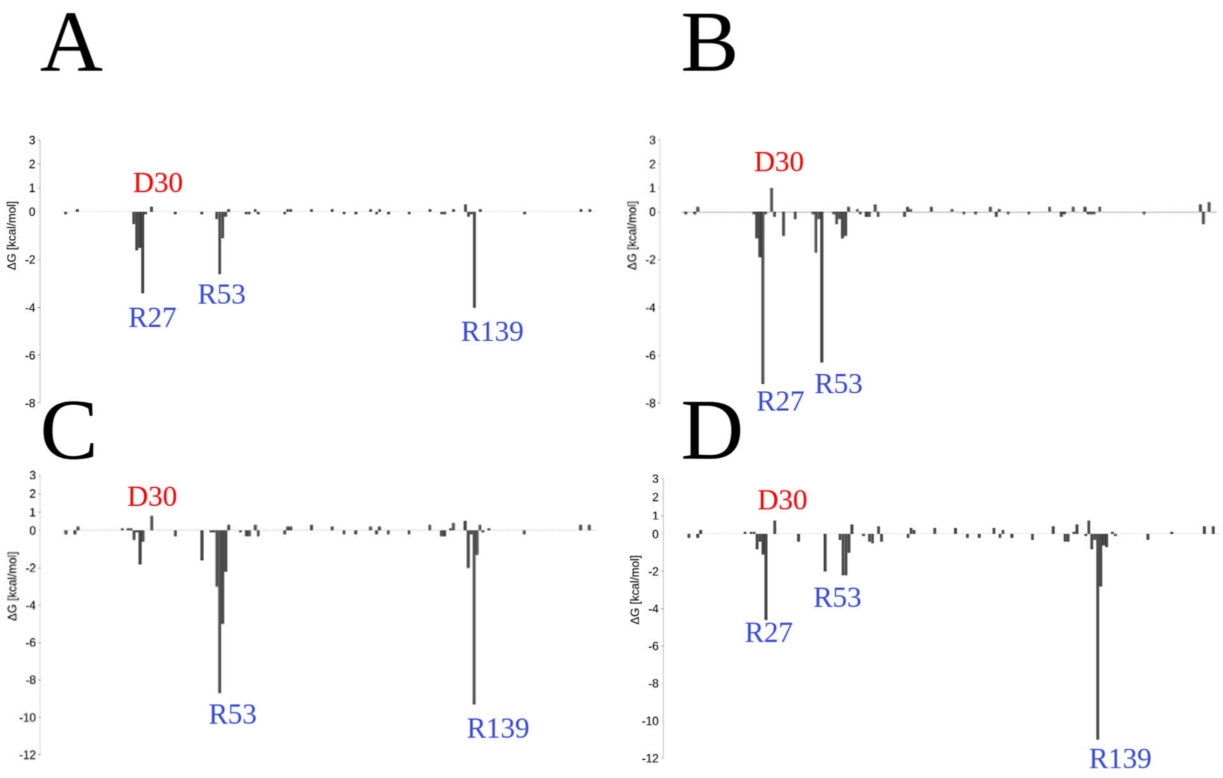

Figure S3. Per residue free energy decomposition for the last nanosecond of the productive MD run for desulfated HS dp2 (A), dp4 (B), dp6 (C) and dp8 (D). In blue and red are amino acid residues showing the most favourable and unfavourable effect on binding energy, respectively.

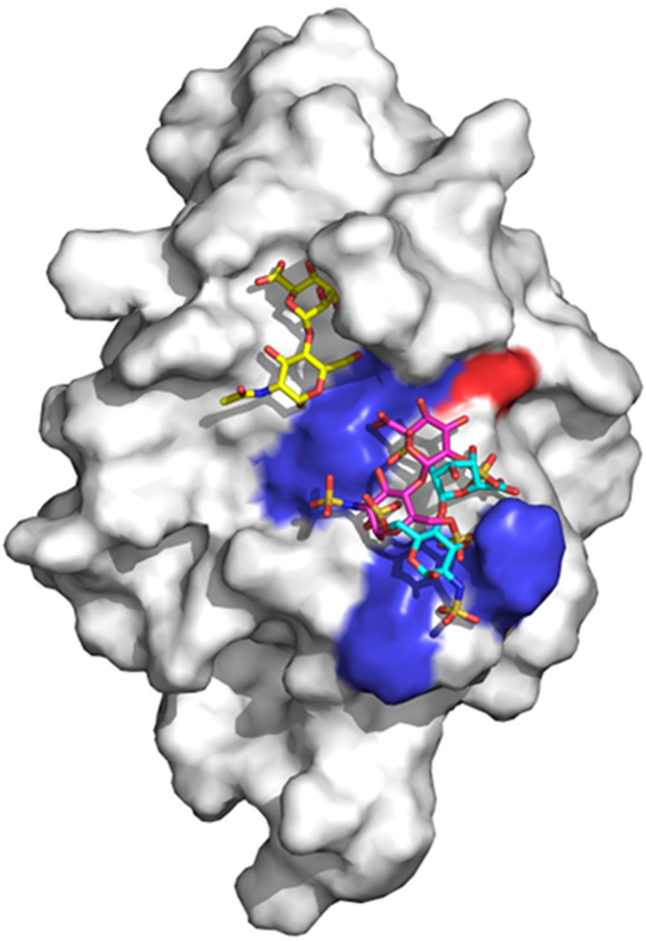

Figure S4. Superimposition of the conformations best scored by MM-GBSA for HP $^{1}C_{4}$ dp2 in cyan, HP $^{2}S_{0}$ dp2 in magenta and desulfated HS dp2 in yellow.

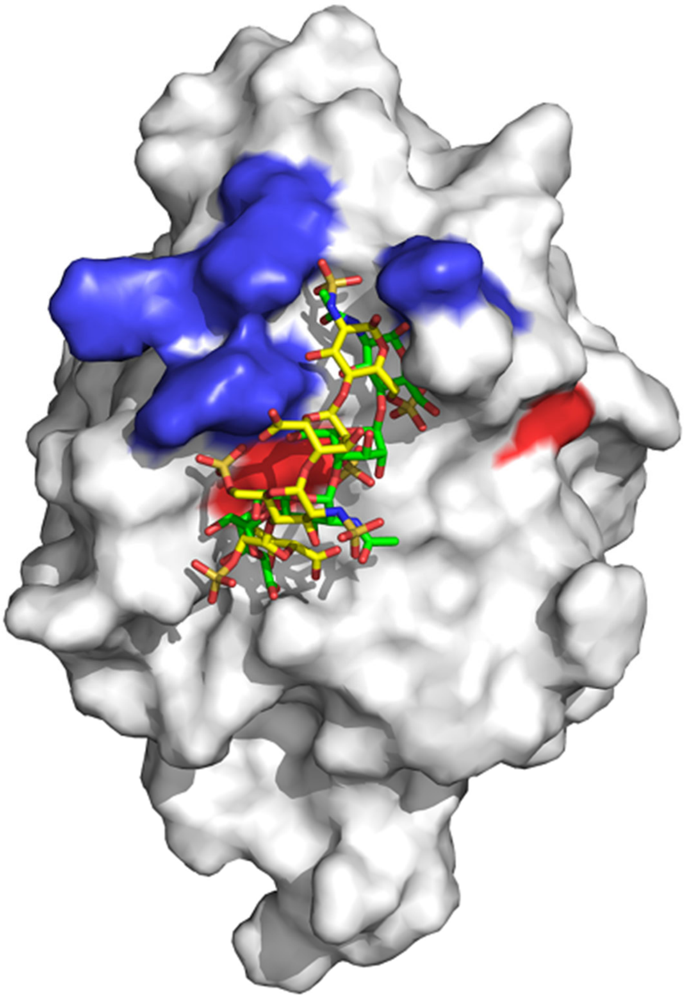

Figure S5. Superimposition of the conformations best scored by MM-GBSA for HP $^{1}C_{4}$ dp4 in green and desulfated HS dp4 in yellow.

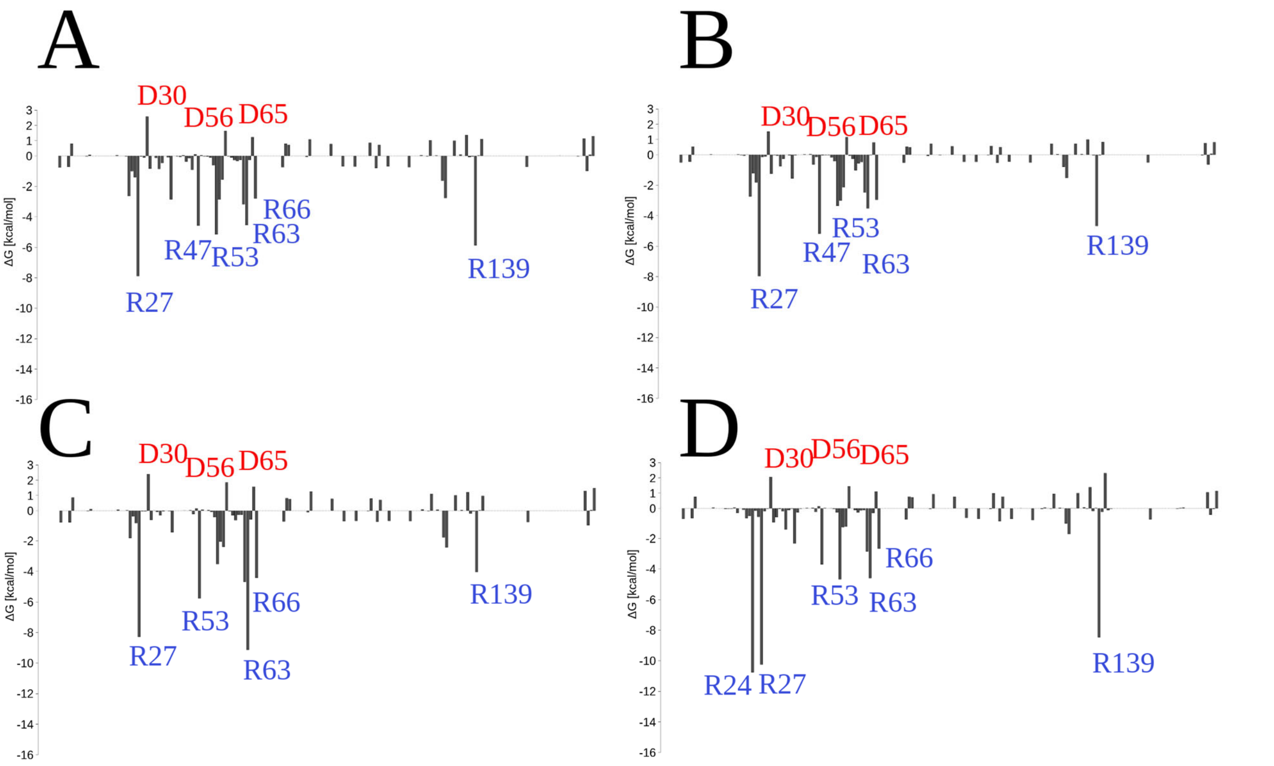

Figure S6. Per residue free energy decomposition for the last nanosecond of the productive MD run for HS with the periodic units GlcNS(6S)-GlcA (A), GlcNS-GlcA (B), GlcNS-IdoA(2S) $^{1}C_{4}$ (C) and GlcNS-IdoA(2S) $^{2}S_{0}$ dp6 (D). In blue and red are amino acid residues showing the most favourable and unfavourable effect on binding energy, respectively.

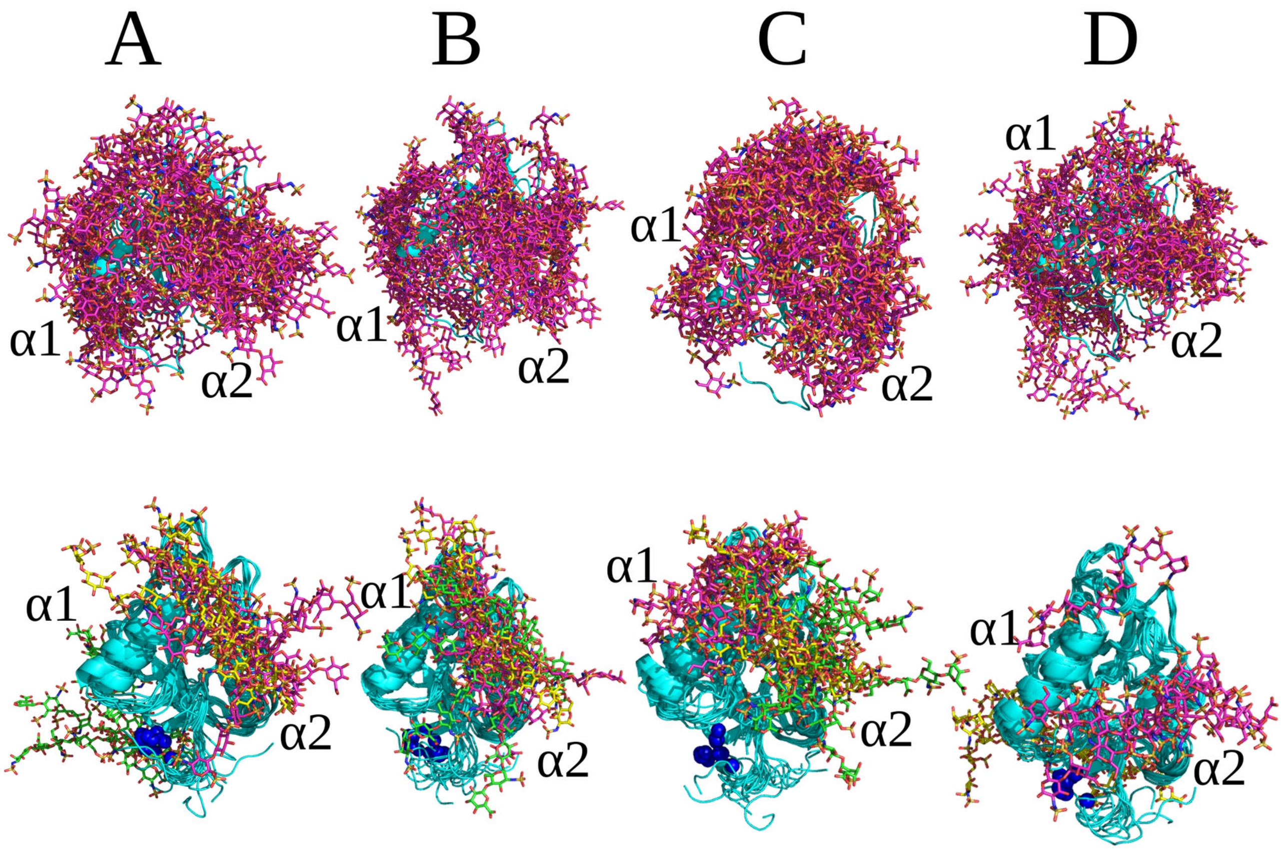

Figure S7. Upper panel: HS top 50 docking poses; lower panel: structures of the complexes obtained from the last frame in MD simulations ; with the repeating units A) GlcNS(6S)-GlcA, B) GlcNS-GlcA, C) GlcNS-IdoA(2S) $^{1}C_4$, D) GlcNS-IdoA(2S) $^{2}S_0$ dp6 (in stick representation, magenta – cluster 1, yellow cluster 2, green cluster 3) with endostatin bound to Zn^{2+} (rendered as a dark blue sphere) in cartoon.

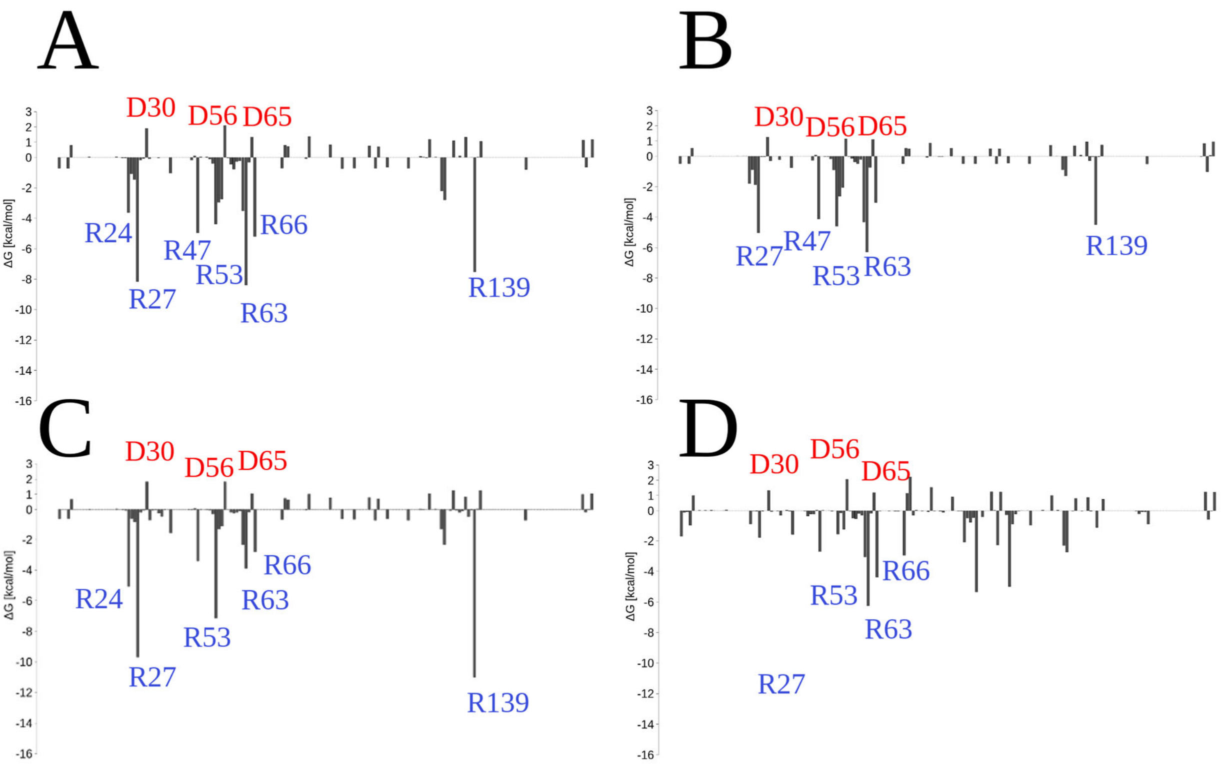

Figure S8. Per residue free energy decomposition for the last nanosecond of the productive MD run for the complex of endostatin bound to Zn^{2+} with HS dp8 with the repeating units GlcNS(6S)-GlcA (A), GlcNS-GlcA (B) GlcNS-IdoA(2S) $^{2}S_0$ (C) and GlcNS-IdoA(2S) $^{1}C_4$ (D). In blue and red are amino acid residues showing the most favourable and unfavourable effect on binding energy, respectively.

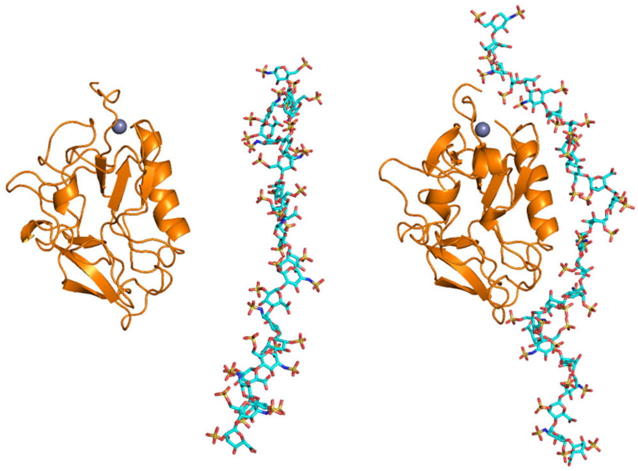

Figure S9. Structure of endostatin-HP dp24 complexes after RS-REMD (left) and refinement procedure (right); proteins are presented in orange, cartoon representation, Zn^{2+} in violet, sphere representation; HP dp24 in cyan, licorice representation.

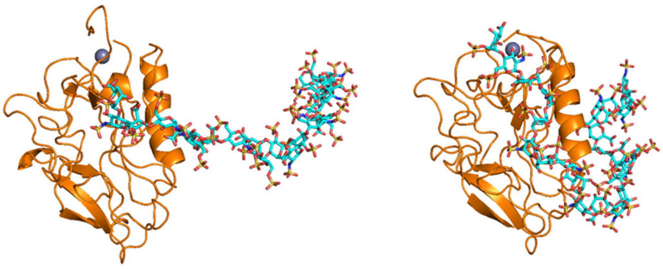

Figure S10. Structure of endostatin-HP dp24 complexes after RS-REMD (left) and refinement procedure (right); proteins are presented in orange, cartoon representation, Zn^{2+} in violet, sphere representation; HP dp24 in cyan, licorice representation.

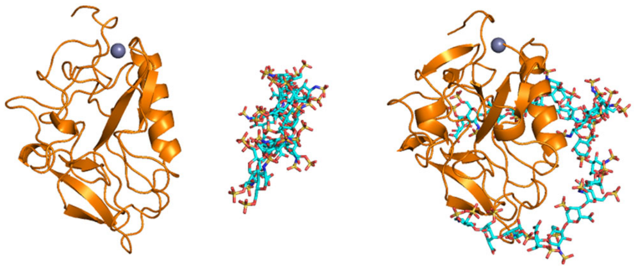

Figure S11. Structure of endostatin-HP dp24 complexes after RS-REMD (left) and refinement procedure (right); proteins are presented in orange, cartoon representation, Zn^{2+} in violet, sphere representation; HP dp24 in cyan, licorice representation.

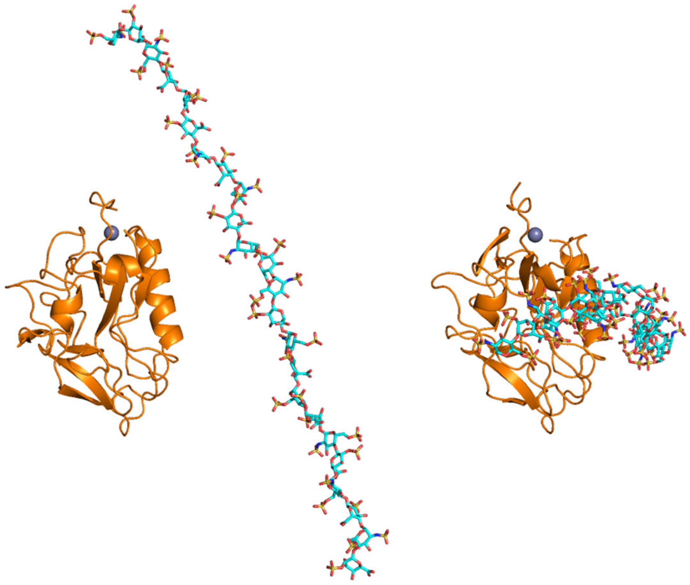

Figure S12. Structure of endostatin-HP dp24 complexes after RS-REMD (left) and refinement procedure (right); proteins are presented in orange, cartoon representation, Zn^{2+} in violet, sphere representation; HP dp24 in cyan, licorice representation.

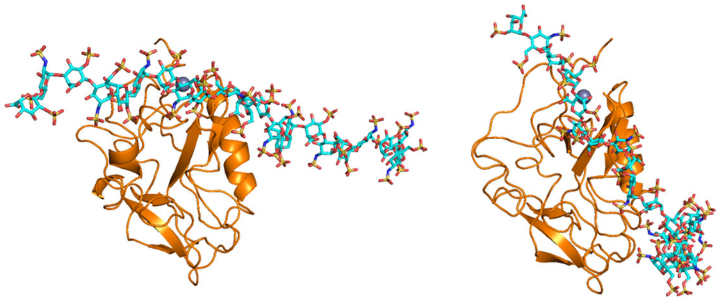

Figure S13. Structure of endostatin-HP dp24 complexes after RS-REMD (left) and refinement procedure (right); proteins are presented in orange, cartoon representation, Zn^{2+} in violet, sphere representation; HP dp24 in cyan, licorice representation.

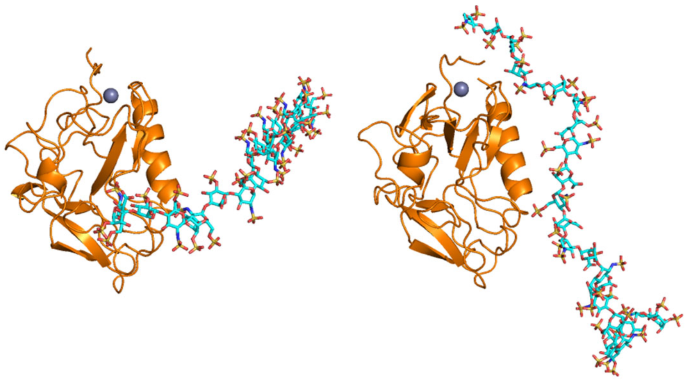

Figure S14. Structure of endostatin-HP dp24 complexes after RS-REMD (left) and refinement procedure (right); proteins are presented in orange, cartoon representation, Zn^{2+} in violet, sphere representation; HP dp24 in cyan, licorice representation.

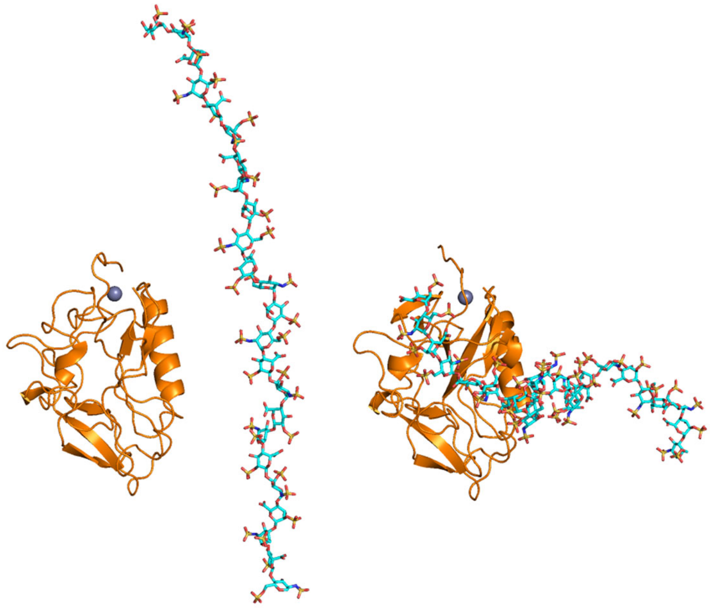

Figure S15. Structure of endostatin-HP dp24 complexes after RS-REMD (left) and refinement procedure (right); proteins are presented in orange, cartoon representation, Zn^{2+} in violet, sphere representation; HP dp24 in cyan, licorice representation.

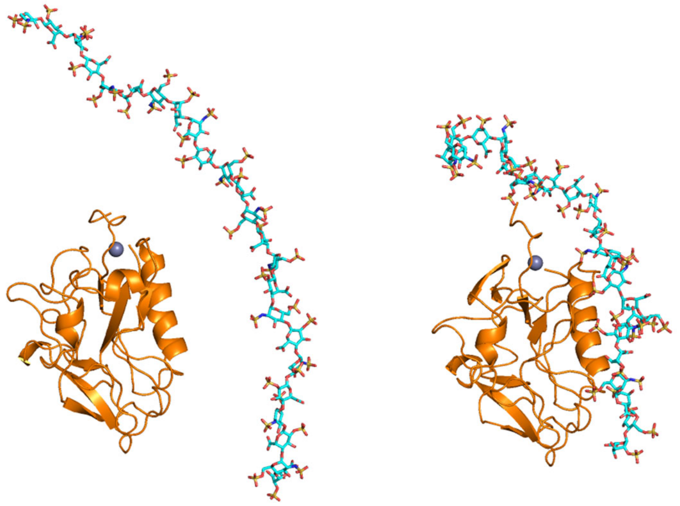

Figure S16. Structure of endostatin-HP dp24 complexes after RS-REMD (left) and refinement procedure (right); proteins are presented in orange, cartoon representation, Zn^{2+} in violet, sphere representation; HP dp24 in cyan, licorice representation.

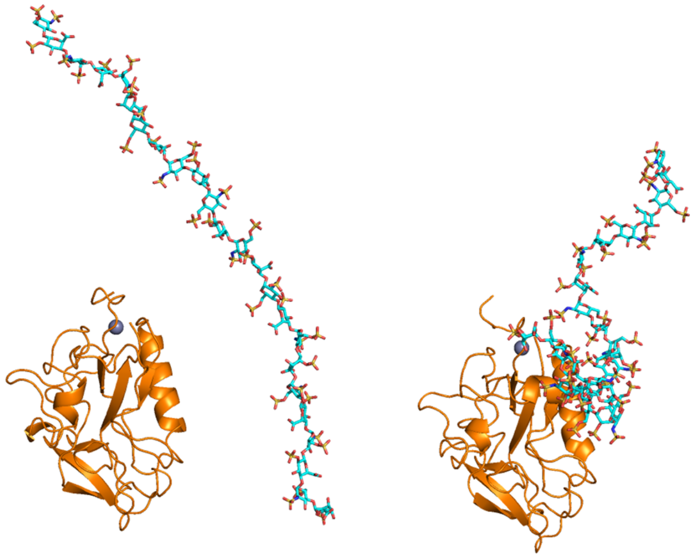

Figure S17. Structure of endostatin-HP dp24 complexes after RS-REMD (left) and refinement procedure (right); proteins are presented in orange, cartoon representation, Zn^{2+} in violet, sphere representation; HP dp24 in cyan, licorice representation.

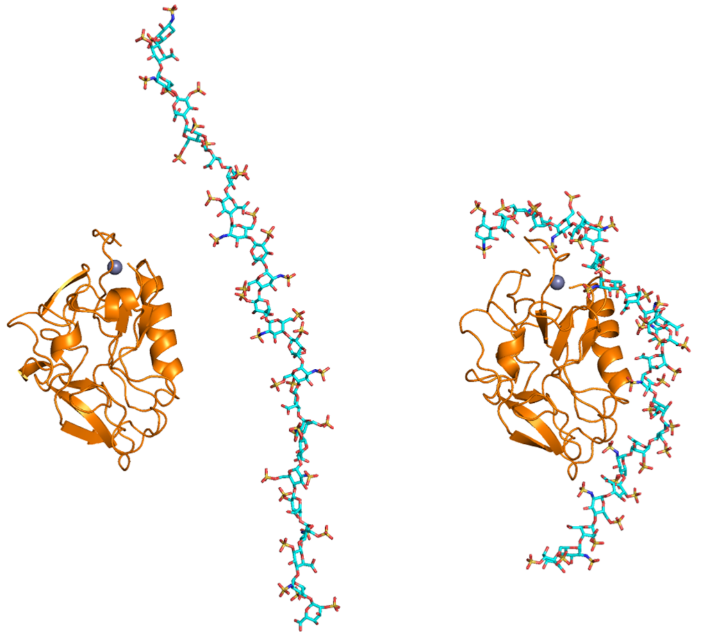

Figure S18. Structure of endostatin-HP dp24 complexes after RS-REMD (left) and refinement procedure (right); proteins are presented in orange, cartoon representation, Zn^{2+} in violet, sphere representation; HP dp24 in cyan, licorice representation.

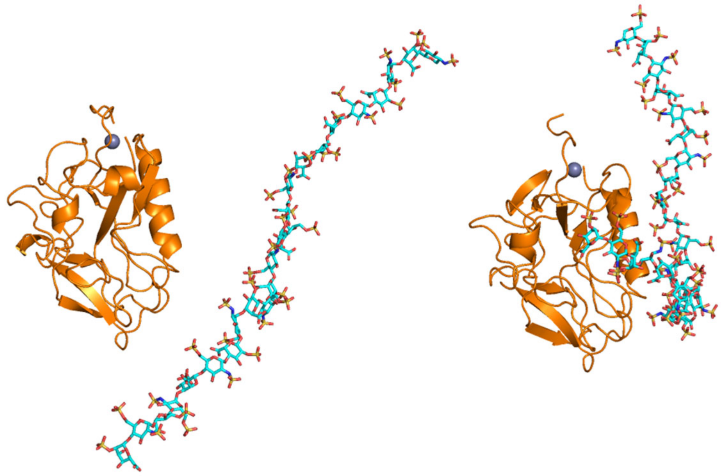

Figure S19. Structure of endostatin-HP dp24 complexes after RS-REMD (left) and refinement procedure (right); proteins are presented in orange, cartoon representation, Zn^{2+} in violet, sphere representation; HP dp24 in cyan, licorice representation.

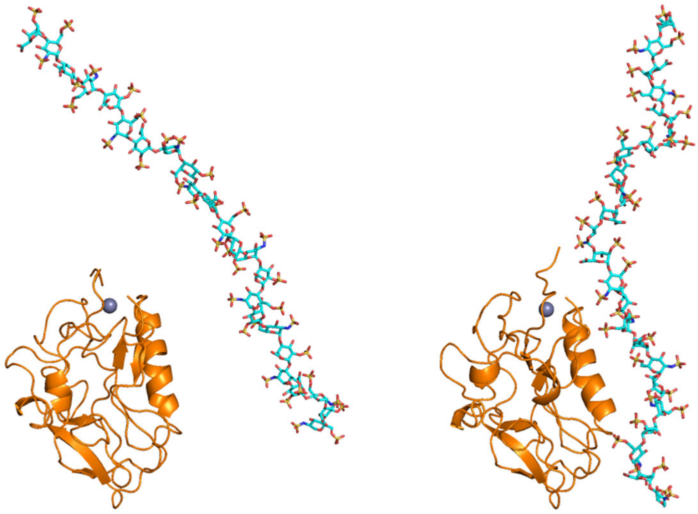

Figure S20. Structure of endostatin-HP dp24 complexes after RS-REMD (left) and refinement procedure (right); proteins are presented in orange, cartoon representation, Zn^{2+} in violet, sphere representation; HP dp24 in cyan, licorice representation.

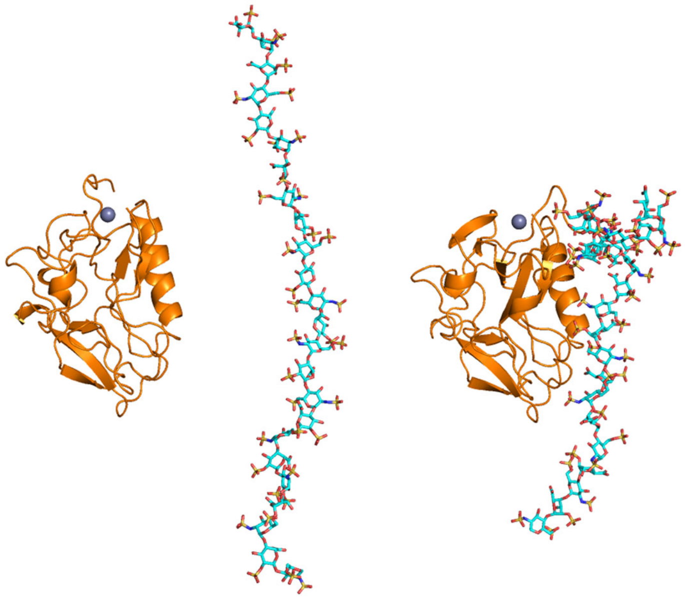

Figure S21. Structure of endostatin-HP dp24 complexes after RS-REMD (left) and refinement procedure (right); proteins are presented in orange, cartoon representation, Zn^{2+} in violet, sphere representation; HP dp24 in cyan, licorice representation.

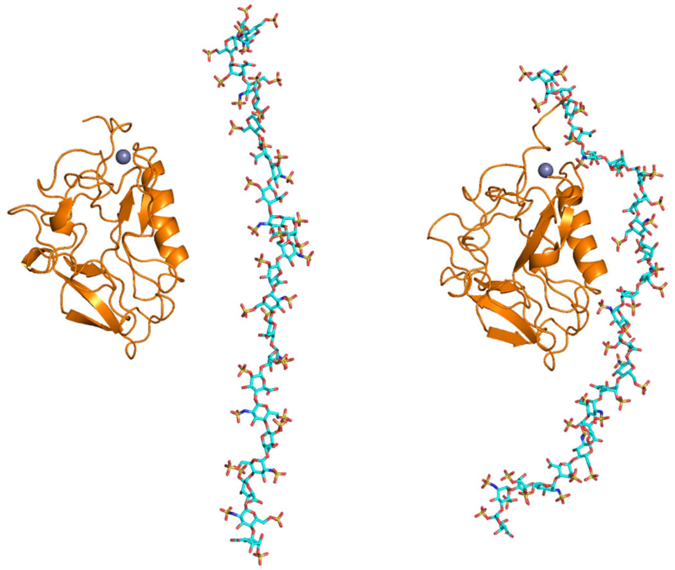

Figure S22. Structure of endostatin-HP dp24 complexes after RS-REMD (left) and refinement procedure (right); proteins are presented in orange, cartoon representation, Zn^{2+} in violet, sphere representation; HP dp24 in cyan, licorice representation.

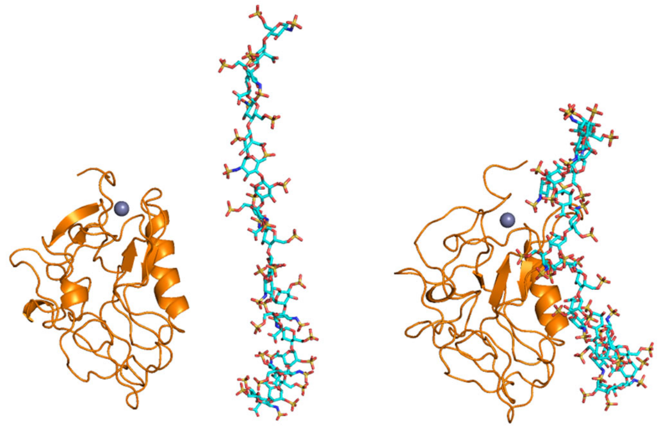

Figure S23. Structure of endostatin-HP dp24 complexes after RS-REMD (left) and refinement procedure (right); proteins are presented in orange, cartoon representation, Zn^{2+} in violet, sphere representation; HP dp24 in cyan, licorice representation.

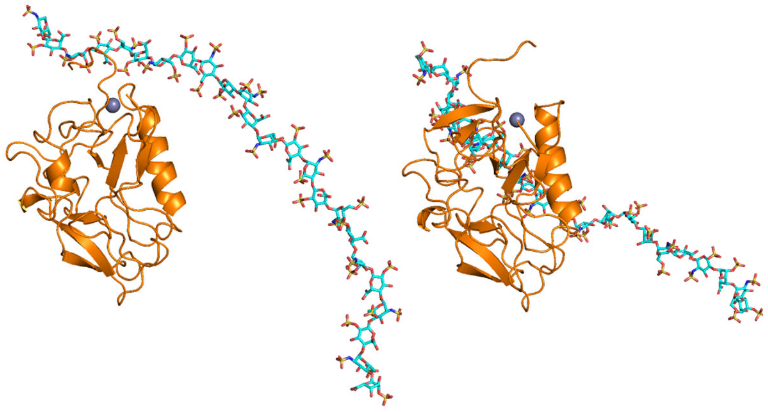

Figure S24. Structure of endostatin-HP dp24 complexes after RS-REMD (left) and refinement procedure (right); proteins are presented in orange, cartoon representation, Zn^{2+} in violet, sphere representation; HP dp24 in cyan, licorice representation.

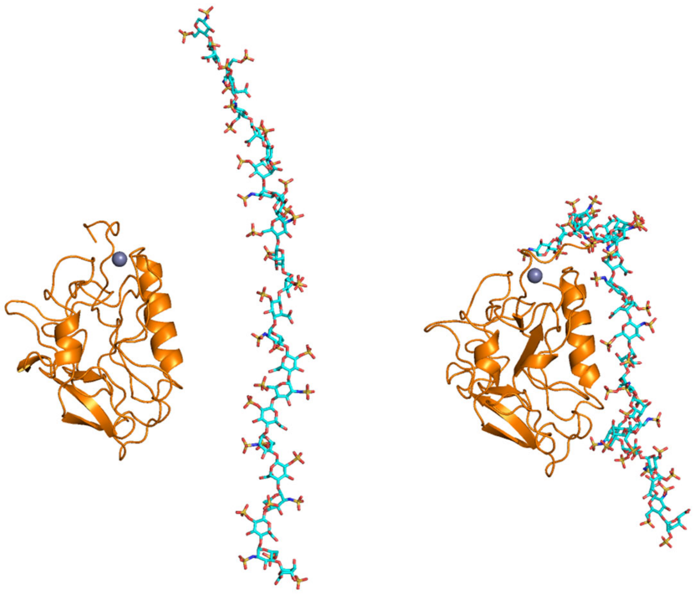

Figure S25. Structure of endostatin-HP dp24 complexes after RS-REMD (left) and refinement procedure (right); proteins are presented in orange, cartoon representation, Zn^{2+} in violet, sphere representation; HP dp24 in cyan, licorice representation.

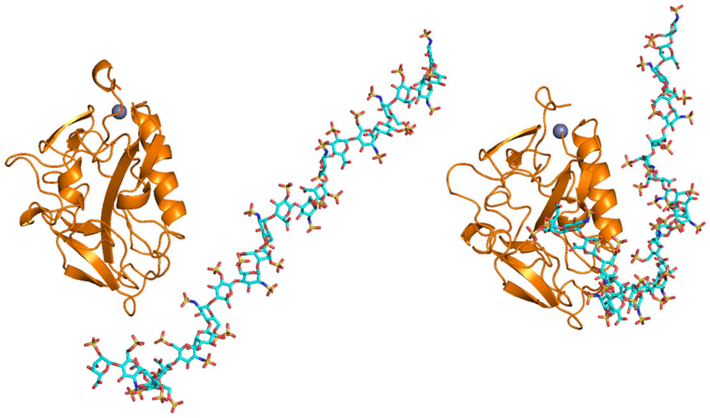

Figure S26. Structure of endostatin-HP dp24 complexes after RS-REMD (left) and refinement procedure (right); proteins are presented in orange, cartoon representation, Zn^{2+} in violet, sphere representation; HP dp24 in cyan, licorice representation.

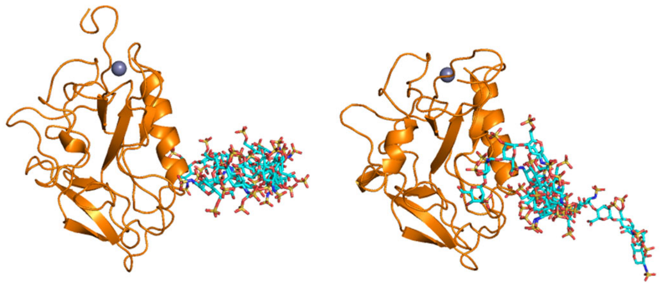

Figure S27. Structure of endostatin-HP dp24 complexes after RS-REMD (left) and refinement procedure (right); proteins are presented in orange, cartoon representation, Zn^{2+} in violet, sphere representation; HP dp24 in cyan, licorice representation.

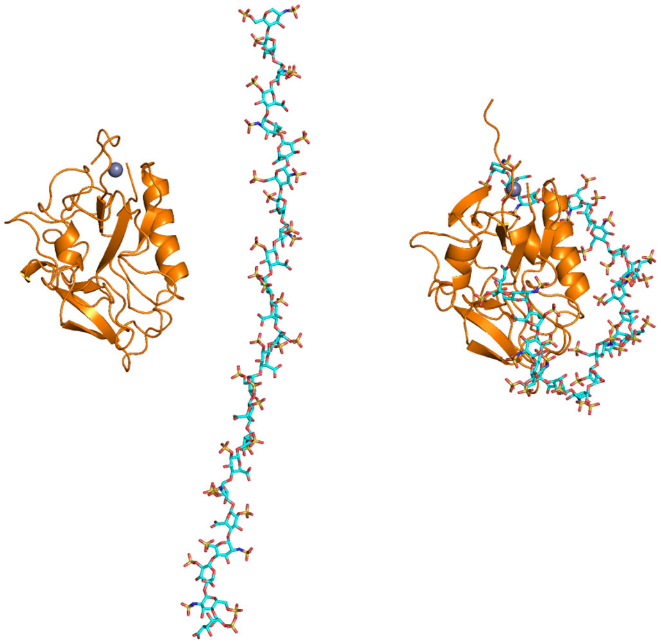

Figure S28. Structure of endostatin-HP dp24 complexes after RS-REMD (left) and refinement procedure (right); proteins are presented in orange, cartoon representation, Zn^{2+} in violet, sphere representation; HP dp24 in cyan, licorice representation.

Table S1. The per residue decomposition analysis for the highest score models (pose 1 and pose 2) of the endostatin-HP dp24 complexes after the refinement procedure.

Pose 1		Pose 2	
Amino acid residue	ΔG, kcal/mol	Amino acid residue	ΔG, kcal/mol
Arg53	-18.0	Arg139	-16.5
Arg63	-15.8	Arg27	-13.8
Arg27	-14.9	Arg53	-12.0
Arg24	-12.2	Arg24	-10.1
Arg128	-11.1	Arg38	-9.6
Arg62	-9.4	Arg4	-9.6
Arg139	-8.5	Arg47	-6.1
Arg129	-6.4	His1	-5.5
Arg66	-5.7	Arg129	-3.4
Arg38	-5.1	Arg62	-3.2
Arg47	-4.4	Arg66	-3.0
Arg4	-3.0	Arg128	-2.8
Ala64	-2.8	Arg63	-2.8
Arg156	-2.8	Gly25	-2.7
His1	-2.8	Lys106	-2.7

Table S2. Hydrogen bond analysis for the highest score pose 1 of the endostatin-HP dp24 complex in the refinement procedure.

**Index**	**Donor**	**Acceptor**	**Population (%)**
1	Arg53 NH	α-L-IdoA(2S) 12 OS	124.7
2	Arg27 NE	α-D-Glc(6S,NS) 11 OS	97.3
3	Arg27 NH_2	α-D-Glc(6S,NS) 11 OS	70.6
4	Arg27 NE	α-D-Glc(6S,NS) 11 O5	69.1
5	Arg63 NE	α-L-IdoA(2S) 20 O6	60.8
6	Arg53 NH_2	α-D-Glc(6S,NS) 11 O6	58.5
7	Ala64 N	α-D-Glc(6S,NS) 21 OS	57.2
8	Arg139 NH	α-L-IdoA(2S) 12 OS	54.0
9	Arg63 NH_2	α-L-IdoAA(2S) 20 O5	49.0
10	Arg53 NH_2	α-L-IdoA(2S) 12 OS	47.6
11	Arg63 NH_2	α-L-IdoA(2S) 20 O6	42.7
12	Arg24 NH_2	α-L-IdoA(2S) 8 OS	41.3
13	Arg53 NH_2	α-L-IdoA(2S) 12 OS	36.2
14	Arg128 NH	α-D-Glc(6S,NS) 19 OS	33.3
15	Arg53 NH_2	α-D-Glc(6S,NS) 11 OS	29.7
16	Arg139 NH	α-L-IdoA(2S) 12 O3	27.6
17	Arg128 NH_2	α-D-Glc(6S,NS) 19 OS	25.6
18	Arg27 NH_2	α-D-Glc(6S,NS) 11 O5	25.0
19	Arg128 NH_2	α-L-IdoA(2S) 18 OS	21.4
20	Ser59 OG	α-D-Glc(6S,NS) 19 OS	21.1
21	Arg27 NH_2	α-D-Glc(6S,NS) 11 OS	21.0

NE is an ε-nitrogen atom; OG is a γ-oxygen atom;
according to AMBER nomenclature 1 is corresponding to reducing end;
O3 is carboxyl oxygen; O5 is pyranose oxygen; O6 is 6-sulfate oxygen; OS is the oxygen belonging to a sulfate group.

Table S3. Hydrogen bond analysis for the highest score pose 2 of the endostatin-HP dp24 complex in the refinement procedure.

Index	Donor	Acceptor	Population, %
1	Arg27 NH	α-D-Glc(6S,NS) 15 O5	48.2
2	Arg27 NH	α-D-Glc(6S,NS) 15 OS	48.1
3	Arg24 NH_2	α-L-IdoA(2S) 18 O6	47.4
4	Arg139 NH	α-L-IdoA(2S) 16 O5	42.2
5	Arg27 N	α-D-Glc(6S,NS) 15 OS	41.8
6	Arg53 NH_2	α-D-Glc(6S,NS) 15 OS	40.8
7	Arg53 NH	α-L-IdoA(2S) 16 O6	38.8
8	Arg139 NH_2	α-L-IdoA(2S) 16 O5	35.7
9	Arg53 NH_2	α-L-IdoA(2S) 16 O5	32.1
10	Ile26 N	α-D-Glc(6S,NS) 15 OS	29.3
11	Arg53 NE	α-D-Glc(6S,NS) 15 OS	28.4
12	Arg139 NH_2	α-L-IdoA(2S) 16 O4	27.8
13	Arg53 NH_2	α-L-IdoA(2S) 16 O6	26.1
14	Arg139 NH_2	α-L-IdoA(2S) 16 O2	24.7
15	Arg4 NH_2	α-D-Glc(6S,NS) 9 OS	22.7
16	Arg38 NH_2	α-L-IdoA(2S) 10 O6	22.1
17	Arg24 NH_2	α-L-IdoA(2S) 16 OS	21.6

18	Arg24 NE	α-L-IdoA(2S) 196 OS	21.3
19	Thr45 OG	α-D-Glc(6S,NS) 191 OS	20.7

NE is an ε-nitrogen atom; OG is a γ-oxygen atom;
according to AMBER nomenclature 1 is corresponding to reducing end;
O2 is 2-sulfate oxygen is carboxyl oxygen; O5 is pyranose oxygen; O6 is 6-sulfate oxygen; OS is the oxygen belonging to a sulfate group.

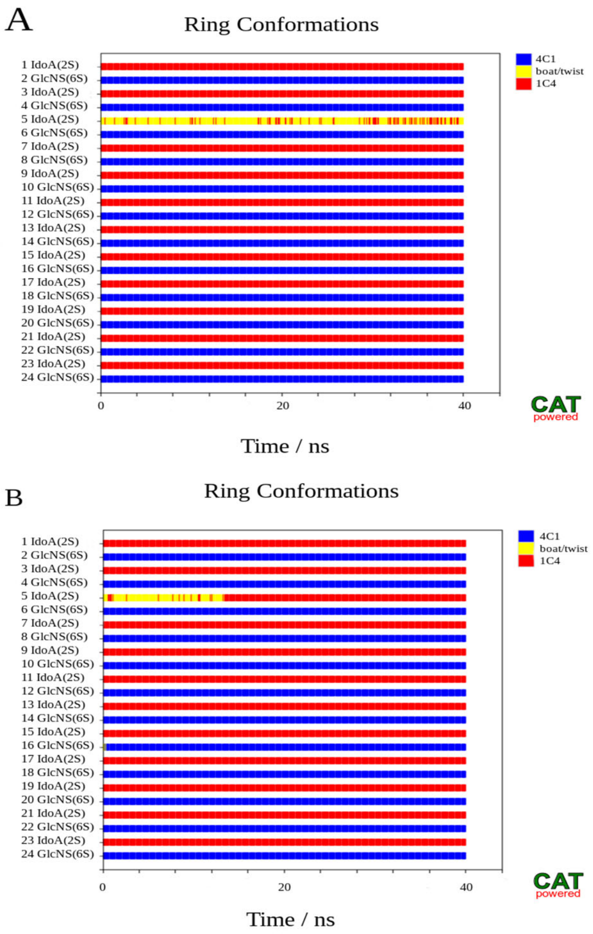

Figure S29. Ring puckering analysis for HP dp24 during the refinement of the endostatin-HP dp24 highest scored pose 1 (A) and pose 2 complexes (B).
